# Do changes in STEC diagnostics mislead interpretation of disease surveillance data in Switzerland? Time trends in positivity, 2007 to 2016

**DOI:** 10.2807/1560-7917.ES.2020.25.33.1900584

**Published:** 2020-08-20

**Authors:** Fabienne Beatrice Fischer, Apolline Saucy, Claudia Schmutz, Daniel Mäusezahl

**Affiliations:** 1 Swiss Tropical and Public Health Institute, Basel, Switzerland; 2 University of Basel, Basel, Switzerland

**Keywords:** STEC/EHEC/VTEC, surveillance, multiplex PCR, diagnostics, notification system

## Abstract

**Background:**

Laboratory-confirmed cases of Shiga toxin-producing *Escherichia coli* (STEC) have been notifiable to the National Notification System for Infectious Diseases in Switzerland since 1999. Since 2015, a large increase in case numbers has been observed. Around the same time, syndromic multiplex PCR started to replace other diagnostic methods in standard laboratory practice for gastrointestinal pathogen testing, suggesting that the increase in notified cases is due to a change in test practices and numbers.

**Aim:**

This study examined the impact of changes in diagnostic methods, in particular the introduction of multiplex PCR panels, on routine STEC surveillance data in Switzerland.

**Methods:**

We analysed routine laboratory data from 11 laboratories, which reported 61.9% of all STEC cases from 2007 to 2016 to calculate the positivity, i.e. the rate of the number of positive STEC tests divided by the total number of tests performed.

**Results:**

The introduction of multiplex PCR had a strong impact on STEC test frequency and identified cases, with the number of tests performed increasing sevenfold from 2007 to 2016. Still, age- and sex-standardised positivity increased from 0.8% in 2007 to 1.7% in 2016.

**Conclusion:**

Increasing positivity suggests that the increase in case notifications cannot be attributed to an increase in test numbers alone. Therefore, we cannot exclude a real epidemiological trend for the observed increase. Modernising the notification system to address current gaps in information availability, e.g. diagnostic methods, and improved triangulation of clinical presentation, diagnostic and serotype information are needed to deal with emerging disease and technological advances.

## Introduction

Infections caused by Shiga toxin (Stx)-producing *Escherichia coli* (STEC) are generally mild and self-limiting or even asymptomatic. However, particularly in children and elderly people, STEC infections can lead to severe gastroenteritis with haemorrhagic diarrhoea and life-threatening conditions, e.g. haemolytic uraemic syndrome (HUS) [1,2].

STEC transmission can occur through the consumption of contaminated food and drinks, or by direct contact with infected individuals or animals shedding the bacterium* [1,3-5]. STEC infections are endemic in Europe, including Switzerland [6,7]. Cases occur sporadically or in outbreaks; a large outbreak attributed to contaminated sprouts occurred in Germany in 2011 [8]. Smaller outbreaks have also been reported, e.g. there was an outbreak in Italy in 2013 and in Romania in 2016, both were suspected to be caused by contaminated dairy products [9,10]. Considering 22 years of population-based data up to 2012, Majowicz et al. estimated in 2014 that STEC leads to an estimated 2.8 million illness cases per year, including 3,800 cases of HUS, globally [11].

The National Notification System for Infectious Diseases (NNSID) of the Swiss Federal Office of Public Health (FOPH) has been receiving all notifications of laboratory-confirmed STEC infections since 1999. Case numbers were generally constant until 2010, with only a few laboratories reporting STEC cases in Switzerland. An increase in cases was observed in 2011 following the outbreak in Germany, before returning to expected yearly fluctuations, and then markedly increasing since 2015 [12]. Given that this increase was observed around the same time as the introduction of syndromic multiplex PCR panels for stool analyses in standard laboratory practice in Switzerland [12], it was hypothesised that these panels were the cause of the increase in notified STEC cases. Traditionally, routine testing of stool samples for bacterial pathogens involved only *Campylobacter* spp., *Salmonella* spp. and *Shigella* spp. using culture-based techniques. With syndromic multiplex PCR panels, stool samples can be tested for up to 22 pathogens, including STEC, in one single run [12,13].

Prior to the gradual introduction of multiplex PCR to the routine diagnostics between 2014 and 2015, STEC was only specifically tested for in Switzerland upon physician request, and this rarely happened. Current testing practice includes the use of small syndromic enteric bacterial panels for testing in patients without a travel history or a larger gastrointestinal panel if travel history is reported on the test order form [7].

 A qualitative assessment found that Swiss laboratory experts uniformly agreed that the increase in STEC case numbers was due to the introduction and increasing use of multiplex PCR panels [7]. We set out to conduct a quantitative investigation as to whether an increase in the STEC testing rate associated with the use of the panels is what led to the increased notification of cases.

Our study assesses the development of the STEC positivity in the Swiss population between 2007 and 2016 using routine laboratory data, and gives insight into the epidemiology and notification numbers of STEC infections in Switzerland.

## Methods

The study uses pre-existing records from the routine work of diagnostic laboratories. Swiss regulatory authorities report 106 authorised or accredited diagnostic laboratories, but not all of them perform STEC diagnostics [14]. Therefore and for feasibility reasons, we decided in 2016 to purposively select 11 diagnostic laboratories to be included in our study. First, the laboratories with the most STEC notifications the year before were selected and their coverage of Swiss regions was checked. For underrepresented regions, we added the top reporting laboratories of these regions to the sample. Our final sample included all regions of Switzerland, and both hospital and private diagnostic laboratories. The organisation of infectious disease diagnostics in Switzerland does not allow for estimating the population covered by the laboratories.

Anonymised, individual-based testing data on STEC from the laboratories’ pre-existing records were received from the FOPH. Data collected comprised all tests performed for STEC between January 2007 and December 2016, including positive and negative test outcomes. Our resulting database included date of test, test result, test method, patient identification number, and patients’ date of birth, sex and canton of residence.

Test records indicating a patient resided outside of Switzerland and those without a conclusive test result were omitted. Duplicate entries, defined as identical values for all variables, and repeated tests were excluded from the analyses. Repeated tests were defined as more than one test performed for the same patient during a single disease episode.

The analysis was planned a priori and was performed using STATA version 14.0 (StataCorp, Texas, United States (US)). A statistical significance level of alpha 0.05 was chosen for all tests and models.

We use the term positivity as the rate of number of positive tests to the total number of tests performed for STEC [15,16]. Positivity was calculated for different demographic groups, test methods, spatial (i.e. patients’ canton of residence) and temporal (annual and seasonal) trends. The main outcome, annual positivity, was age- and sex-adjusted using direct standardisation with the sample population (2007–2016) as reference population.

We calculated odds ratios (ORs) for the association between test result and test year, test month, season, a discrete time trend variable, sex, age group, laboratory, test method and greater region using univariable logistic regression. Season was modelled using a sine and cosine function with an annual period. The time trend was a discrete variable constructed of all test months combining the test month and test year variables. The greater regions correspond to the seven regions of Switzerland as specified by the Nomenclature of Territorial Units for Statistics (NUTS)-2. Categories with most observations were chosen as reference categories, except for the seasonality (first month of the year).

We defined a multivariable mixed-effect logistic regression model a priori, independent of the outcome of the univariable regression, to calculate adjusted ORs (aORs). The model’s explanatory variables included sex, age group, seasonality, time trend, greater region, diagnostic test method, and an interaction term for sex and age group. Laboratories were included as a random effect variable to account for clustering. Clustering on patient level (same identification number) was omitted.

Finally, we compared the fully adjusted multivariable model to a multivariable model without adjustment for test method in order to validate the results and ensure the consistency of the time trend, independently from the diagnostic method.

Based on multivariable regression results, we computed predicted probabilities for a positive test result, and plotted them for direct visualisation and comparison of categories and models.

We also performed a sensitivity analysis, omitting laboratories not providing data for the entire study period to account for the impact of the missing data. For relevant figures, both the complete dataset referring to data from all 11 laboratories, and the reduced dataset, referring to only the laboratories providing data for the entire study period, are shown.

### Ethical statement

The study was conducted under the Epidemics Act (SR 818.101). The study team received anonymised laboratory data from the FOPH, who had received already-anonymised data directly from the laboratories. Other data (notification data, population statistics) are publicly available from the FOPH or the Swiss Federal Statistical Office.

## Results

### Number of test records and STEC-positives

The 11 participating laboratories provided 91,685 STEC test records, of which, 1,366 were positives. Five laboratories (laboratories B, G, H, I and J) provided data for the entire study period of 2007 to 2016 (n = 61,916). Three laboratories (C, D and F) started performing STEC testing between 2014 and 2015 with the introduction of multiplex PCR panels, two laboratories (A and E) could not extract all data requested because of changes in their data storage system and one laboratory (K) did not specify a reason for missing years of data. Sensitivity analyses omitting laboratories not providing data for the entire study period showed that observed trends were robust. Therefore, the complete dataset without omission is presented and discussed. Relevant figures show the data with and without omission.

Following our exclusion criteria, 1,407 records, including 22 positives, were excluded. Further, 71 records (3 positives) with missing sex or age, 1,110 duplicated entries (31 positives) and 3,054 repeated tests (96 positives) were excluded. The final dataset comprised 86,043 records, of which, 1,149 were positives.

**Figure 1:**
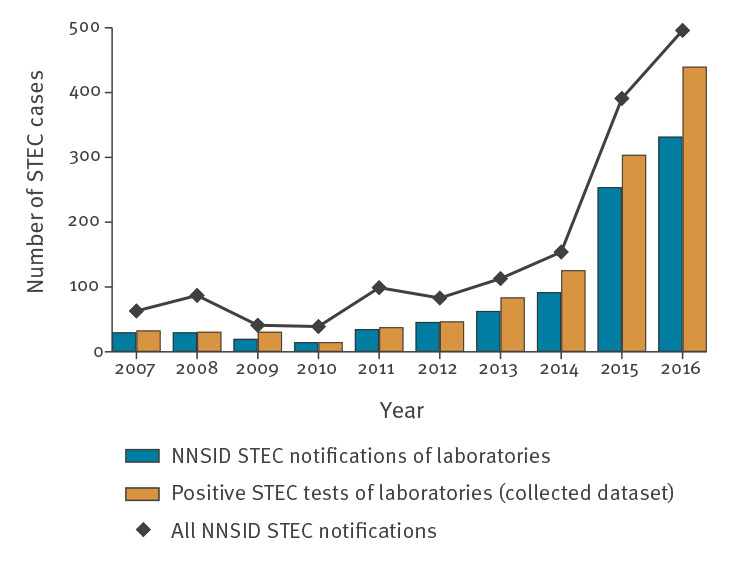
Number of STEC notifications to NNSID versus number of positive STEC tests of 11 diagnostic laboratories, and total number of STEC notifications to NNSID per year, Switzerland, 2007–2016

NNSID: National Notification System for Infectious Diseases; STEC: Shiga toxin-producing *Escherichia coli*.

Figure 1 shows the number of notified STEC cases in the NNSID and in our dataset. In concert, the laboratories selected for this study reported 61.9% of all cases registered in the NNSID between 2007 and 2016 (range 39.4% in 2011 to 73.2% in 2009).

### Characteristics of the tested and STEC-positive population

Median age of the tested population increased significantly from 30 to 43 years between 2007 and 2016 (test for trend: p < 0.01, Supplementary Table S1). The proportion of females tested in this period was 55.6% on average and remained level throughout the test years. The median age of the tested population differed significantly between laboratories (Kruskal-Wallis test: p < 0.01, range: 27–55, overall median: 40; data not shown) and greater regions (Kruskal-Wallis test: p < 0.01, range: 37–44; data not shown).

Similarly, among the STEC-positive population, the median age increased significantly from 2007 to 2016, while the proportion of females remained stable (test for trend: p < 0.01, Supplementary Table S1). Median age differed significantly between laboratories (Kruskal-Wallis test: p < 0.01, range: 2.5–55, overall median: 36; data not shown), but not between regions (Kruskal-Wallis test: p = 0.399, range: 34–68; data not shown). The average number of disease episodes per person was one, with a maximum of four for 122 persons (data not shown).

### Laboratories, diagnostic methods and greater regions

The variables laboratory, greater region and test method were strongly correlated (see Supplementary Figure S2).

The diagnostic methods performed included multiplex PCR (66.5%, n = 57,168), antigen test (26.3%, n = 22,588), single PCR, i.e. PCR panels targeting STEC/pathogenic *E. coli* only (7.3%, n = 6,247), and culture-based diagnostics (< 0.1%, n = 24). Sixteen (< 0.1%) tests did not have a test method specified (outsourced tests). Multiplex PCR panels used were mainly BD MAX (normal or extended) Enteric Bacterial Panel (BD, Franklin Lakes, US) (51.6%), xTAG Gastrointestinal Pathogen Panel (Luminex, Austin, US) (36.1%), BioFire FilmArray Gastrointestinal Panel (BioFire, Salt Lake City, US) (5.9%) and Seegene, not specified whether Allplex Gastrointestinal Panel or Seeplex Diarrhoea ACE Detection (Seegene, Seoul, South Korea) (4.6%). All available information on the test methods applied as reported by the laboratories is presented in Supplementary Table S2.

**Figure 2:**
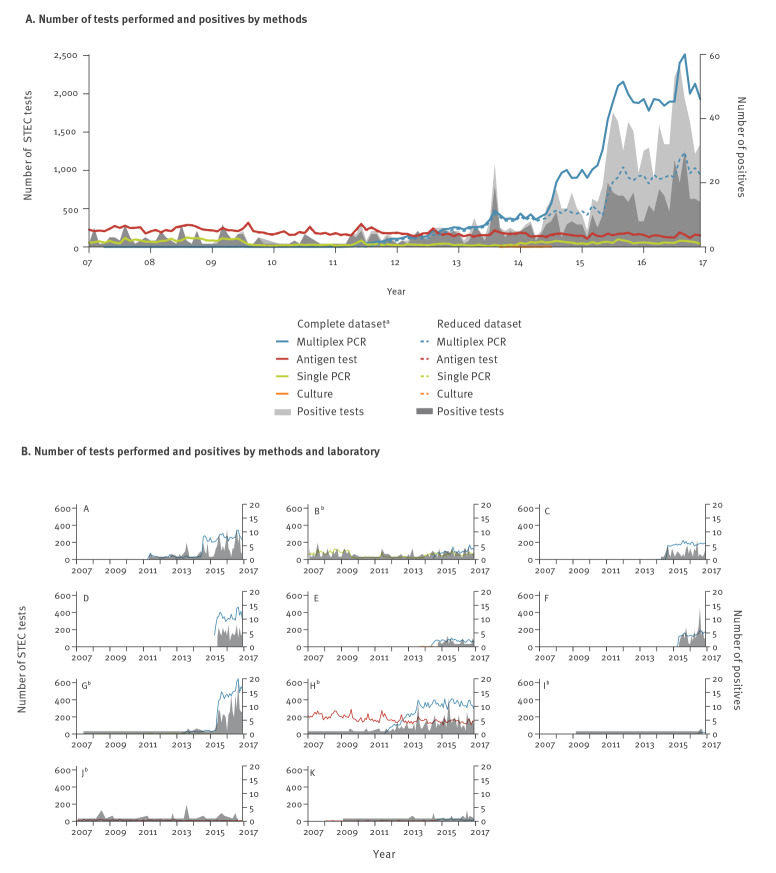
Total number of STEC tests performed and number of positive tests by test method (A) and by laboratory (B), 11 diagnostic laboratories, Switzerland, 2007–2016

The number of tests performed using the antigen test, single PCR or culture remained stable between 2007 and 2016, while the number of multiplex PCR panels performed increased by 42% (Figure 2A). The five laboratories providing data for the entire study period were using single PCR or antigen tests before the introduction of multiplex PCR (Figure 2B). Only one of these five laboratories continued using primarily antigen tests for the entire study period.

STEC: Shiga toxin-producing *Escherichia coli*.

^a^ Complete dataset refers to data from all 11 laboratories, while reduced dataset refers to only the five laboratories providing data for the entire study period.

^b^ The five laboratories providing data for the entire study period. For laboratories G and I, the numbers starting at 2007 are too small to appear on the figure.

### Positivity

**Figure 3:**
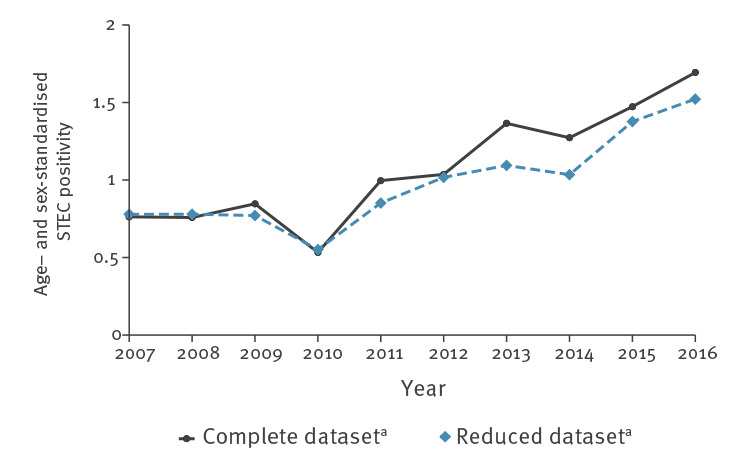
Age- and sex-standardised positivity of STEC testing, 11 diagnostic laboratories, Switzerland, 2007–2016

The number of tests for STEC increased sevenfold from 2007 to 2016 (3,711 to 26,639) while the number of positive test results increased 13-fold (33 to 440). The age- and sex-standardised positivity of STEC testing increased from 0.8% in 2007 to 1.7% in 2016 (Figure 3).

STEC: Shiga toxin-producing *Escherichia coli*.

^a^ Complete dataset refers to data from all 11 laboratories, while reduced dataset refers to only the five laboratories providing data for the entire study period.

Positivity increased for all age categories. The positivity calculated over the entire study period was highest for children aged 1–4 years (192/8,855, 2.2%) and increased from 1.4% (11/809) in 2007 to 2.9% (51/1,734) in 2016. The largest relative increase was in individuals ≥ 80 years of age, from no case among 146 in 2007 to 1.8% (45/2,449) in 2016.

The overall positivity is similar for men (518/38,209, 1.4%) and women (631/47,834, 1.3%) and increased from 0.6 (11/1,705) and 1.1% (22/2,006) to 1.7% (198/11,682) and 1.6% (242/14,957), respectively, from 2007 to 2016.

**Figure 4:**
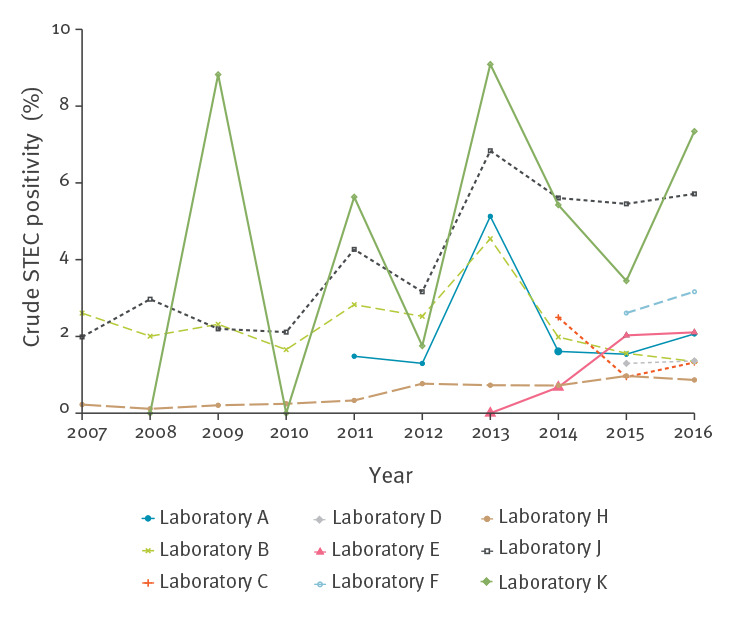
STEC positivity by laboratory, nine diagnostic laboratories^a^, Switzerland, 2007–2016

The positivity and trend in positivity differed across laboratories (Figure 4). The overall positivity ranged from 0.6% (245/38,796) to 5.8% (7/121). There were large fluctuations in positivity for some laboratories because of small testing numbers.

STEC: Shiga toxin-producing *Escherichia coli*.

^a^ Two of the 11 laboratories comprising the dataset are not shown because of the large fluctuations in positivity (range: 0–50%) because of small testing numbers.

Positivity further differed by test method. We did not calculate the positivity of culture-based tests because there were few observations and because of our exclusion process for repeated tests (observations excluded if used as confirmation tests). The positivity across all test years was highest for tests using single PCR (147/6,247, 2.4%) and lowest for the antigen test (129/22,588, 0.6%); positivity of multiplex PCR panels was at 1.5% (870/57,168). The positivity of multiplex PCR increased from 1.1% (80/7,617) in 2014 to 1.7% (418/24,190) in 2016. In contrast, the positivity of single PCR and antigen tests started to decrease in 2014 and 2015 respectively, after PCR peaking at 4.3% (11/256) in 2013 and antigen tests at 1.4% (27/1,896) in 2014.

### Predictors of a positive diagnostic test result

The univariable regressions showed a marginal but significant trend for the time trend variable (OR: 1.003, p < 0.01, Table). All test years except 2013 showed decreased odds for a positive test outcome compared with the reference year 2016. All calendar months except July have smaller odds for a positive test outcome than the reference month August.

**Table:** Odds ratios for a positive STEC test result of the uni- and multivariable logistic regression models, Switzerland, 2007–2016 (n = 86,043)

Variable	n	OR	95% CI	aOR^a^	95% CI
**Age group (year)**					
Under 1	2,915	0.97	0.67–1.40	1.28	0.72–2.28
1–4	8,855	1.88^b^	1.56–2.27	3.38^b^	2.56–4.45
5–9	2,593	1.80^b^	1.34–2.43	1.66^c^	1.07–2.58
10–19	5,898	1.03	0.79–1.35	1.03	0.71–1.49
20–39	21,971	Ref	NA	Ref	NA
40–59	19,404	1.00	0.84–1.20	1.03	0.81–1.31
60–79	17,685	1.10	0.92–1.32	1.05	0.82–1.34
Over 79	6,722	1.14	0.89–1.45	1.11	0.81–1.52
**Sex**					
Male	38,209	1.03	0.91–1.16	0.93	0.72–1.20
Female	47,834	Ref	NA	Ref	NA
**Male, age group (year)**					
Under 1	1,582	NA	NA	1.14	0.52–2.47
1–4	4,962	NA	NA	0.92	0.62–1.36
5–9	1,325	NA	NA	1.23	0.67–2.27
10–19	2,827	NA	NA	1.14	0.66–1.95
20–39	9,080	NA	NA	Ref	NA
40–59	8,833	NA	NA	1.02	0.70–1.47
60–79	7,408	NA	NA	1.27	0.88–1.84
Over 79	2,192	NA	NA	1.17	0.69–1.95
**Greater region**					
Lake Geneva region	15,526	0.79^d^	0.66–0.93	1.20	0.89–1.60
Espace Mittelland	20,000	Ref	NA	Ref	NA
Northwestern Switzerland	15,273	0.39^b^	0.32–0.49	0.69^d^	0.53–0.89
Zurich	14,439	0.79^d^	0.66–0.94	0.75^c^	0.58–0.98
Eastern Switzerland	6,474	0.70^d^	0.55–0.90	0.88	0.67–1.16
Central Switzerland	10,015	0.90	0.74–1.09	0.92	0.70–1.21
Ticino	1,008	0.74	0.43–1.30	1.30	0.73–2.32
**Test method**					
Multiplex PCR	57,168	Ref	NA	Ref	NA
Antigen test	22,588	0.37^b^	0.31–0.45	0.34^b^	0.26–0.44
Single PCR	6,247	1.56^b^	1.31–1.86	2.31^b^	1.55–3.45
Culture	24	NC	NC	NC	NC
**Time trend**	**86,043**	**1.00^b^**	**1.00–1.01**	**1.00^c^**	**1.00–1.01**
**Test month**					
January	6,040	0.50^b^	0.37–0.68	NA	NA
February	5,529	0.59^d^	0.44–0.80	NA	NA
March	6,137	0.58^b^	0.43–0.77	NA	NA
April	5,872	0.76^c^	0.58–0.99	NA	NA
May	6,357	0.69^d^	0.53–0.90	NA	NA
June	7,084	0.77^c^	0.60–0.99	NA	NA
July	7,321	1.08	0.86–1.35	NA	NA
August	9,154	Ref	NA	NA	NA
September	8,919	0.68^d^	0.54–0.87	NA	NA
October	8,098	0.78^c^	0.61–0.99	NA	NA
November	8,000	0.71^d^	0.55–0.91	NA	NA
December	7,532	0.62^b^	0.47–0.81	NA	NA
**Seasonality**					
sin((d*2*π)⁄T)	86,043	0.84^b^	0.77–0.91	0.89^b^	0.82–0.98
cos((d*2*π)⁄T)	86,043	0.83^b^	0.76–0.90	0.81^c^	0.75–0.89
**Test year**					
2007	3,711	0.53^d^	0.37–0.76	NA	NA
2008	3,978	0.47^b^	0.32–0.67	NA	NA
2009	3,421	0.54	0.38–0.79	NA	NA
2010	2,536	0.35^b^	0.21–0.59	NA	NA
2011	3,393	0.67^c^	0.48–0.94	NA	NA
2012	4,483	0.63^d^	0.47–0.85	NA	NA
2013	6,152	0.82	0.65–1.04	NA	NA
2014	10,246	0.74^d^	0.61–0.90	NA	NA
2015	21,484	0.85^c^	0.74–0.99	NA	NA
2016	26,639	Ref	NA	NA	NA
**Laboratory**					
A	8,712	2.98^b^	2.44–3.64	NA	NA
B	8,861	3.15^b^	2.59–3.83	NA	NA
C	5,102	2.09^b^	1.60–2.75	NA	NA
D	7,181	2.13^b^	1.68–2.70	NA	NA
E	2,197	2.84^b^	2.02–4.00	NA	NA
F	2,904	4.80^b^	3.75–6.16	NA	NA
G	9,852	2.86^b^	2.36–3.48	NA	NA
H	38,796	Ref	NA	NA	NA
I	121	9.66^b^	4.46–20.94	NA	NA
J	1,438	6.14^b^	4.55–8.28	NA	NA
K	879	8.09^b^	5.81–11.27	NA	NA

aOR: adjusted odds ratio; CI: confidence interval; NA: not applicable; NC: not calculated; OR: odds ratio; Ref: reference group for comparison; STEC: Shiga toxin-producing *Escherichia coli*.

^a^ Adjusted for sex, age group, method, temporal trend and seasonality (refer to Supplement S1 and Supplementary Figure S1 for details). Interaction between age and sex. Random effect of laboratory.

^b^ p < 0.001.

^c^ p < 0.05.

^d^ p < 0.01.

^e^ The estimates for culture-based tests could not be calculated because of small testing numbers.

The age groups 1 to 4 years and 5 to 9 years were almost twice as likely to have a positive test outcome (OR 1.88, p < 0.001 and OR 1.80, p < 0.001) than the reference category 20 to 39 years. No difference was observed between sexes.

Compared with multiplex PCR panels, the use of the antigen test had a 63% lower probability to generate a positive test outcome (OR 0.37, p < 0.001 ), while the use of single PCR showed 56% higher chance for a positive test outcome (OR 1.56, p < 0.001).

The ORs and significance levels from the fully adjusted multivariable model, presented in the Table, varied only marginally from the univariable models and do not alter the interpretation; therefore, they are not commented here.

Predicted probabilities based on the fully adjusted multivariable model showed an increasing time trend for all test methods and regions.

**Figure 5:**
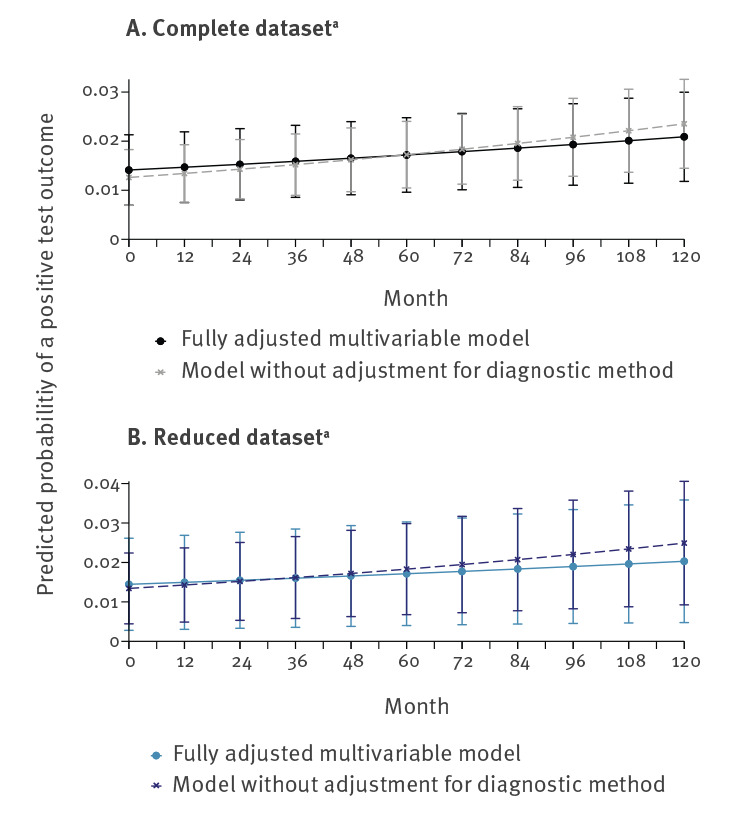
Predicted probability for a positive STEC test outcome for the fully adjusted multivariable model and the model excluding adjustment for test method for the complete (A) and reduced (B) dataset, 11 diagnostic laboratories, Switzerland, 2007–2016

Comparison of the fully adjusted multivariable model to a multivariable model excluding the adjustment for test method showed increasing predicted probabilities for both models, but with a smaller slope for the fully adjusted model (Figure 5).

STEC: Shiga toxin-producing *Escherichia coli*.

^a^ Complete dataset refers to data from all 11 laboratories, while reduced dataset refers to only the five laboratories providing data for the entire study period.

## Discussion

We investigated the apparent epidemic increase of STEC infections seen in the rise of case notifications in the Swiss NNSID. We calculated positivity as the rate of all positive diagnostic STEC tests to the total number of STEC tests performed. The 11 laboratories in our study reported almost two-thirds (61.9%) of all STEC cases in the NNSID between 2007 and 2016. Positivity increased since 2007.

### Culture-independent diagnostic tests for STEC

The increase of STEC cases in Switzerland coincides with the introduction of multiplex PCR panels as a new diagnostic method for STEC detection. The impact of changes in diagnostic approaches on public health surveillance has been highlighted before, especially concerning the switch from culture-dependent to culture-independent diagnostics for food-borne diseases [17-19]. This switch is particularly important for STEC, as the case definitions for STEC in the European Union/European Economic Area (EU/EEA) and Switzerland are not limited to culture-confirmed cases, but include the detection of the Stx1 or Stx2 antigen or their respective genes [20]. Increases in STEC notifications in Ireland were explained by the shift from culture-dependent to culture-independent diagnostic methods; the latter showing higher sensitivity and ability to detect non-O157 STEC [21,22].

The 11 Swiss diagnostic laboratories included in our study switched to culture-independent methods for STEC detection before 2007; hence, the impact thereof cannot be assessed using our data.

### Considerations when using multiplex PCR panels for STEC diagnosis

The introduction of multiplex PCR panels for gastrointestinal pathogens is the next paradigm shift in diagnostics for food-borne diseases after switching to culture-independent tests.

In most of our study laboratories, the use of multiplex PCR panels as routine diagnostic methods was introduced between 2011 and 2015. Since then, multiplex panels comprise the largest proportion of all diagnostic tests performed for STEC and have led to an increase in test numbers. The increase in test volume, resulting in more positives notified, originates from a larger proportion of the population being automatically screened for STEC. This screening happens for two reasons: (i) the testing for a specific gastrointestinal pathogen, e.g. *Campylobacter* spp., now also implicitly leads to a STEC test or (ii) the physician orders a gastrointestinal panel when the patient presents with diarrhoea, i.e. syndromic testing. Previously, a test for STEC was predominantly ordered if the patient was a child and/or reported a bloody stool and/or reported a history of travel because of higher probabilities to develop severe complications such as HUS [23-25]. We hypothesised that if the increase in new STEC cases was a result of the introduction of multiplex PCR only (leading to less targeted screening) there would be a decrease in positivity because of a lower pre-test probability for a positive test outcome. But this decrease in positivity is not reflected in our data. Instead, the increase in STEC cases is disproportionally higher compared with the increase in test volume, resulting in the observed increase in positivity.

Part of the increased testing could also stem from a change in physicians’ test-ordering behaviour following the raising of public awareness for STEC infections. However, laboratory experts reported that tests specifically for STEC are rarely ordered by treating physicians [7]. Therefore, STEC tends to largely be an unintentional finding and its clinical relevance for the individual patient may be arguable. Questions on reporting to the patient and appropriate treatment, see Davis et al. [26], and mandatory notification still need to be addressed.

Furthermore, using multiplex PCR increases the number of cases found because of the higher sensitivity of PCR compared with other conventional diagnostic methods, and the increased probability of detecting co-infections [27-30]. A study among staff members of meat-processing companies in Switzerland found 3.5% asymptomatic carriers of STEC [31]. Assuming a similar prevalence of asymptomatic carriers in the general population and the possibility that such asymptomatic STEC carriers become infected with another diarrhoeagenic pathogen, multiplex PCR would detect both the symptom-causing pathogen and the asymptomatic STEC co-infection.

While it is clear that changes in the diagnostic landscape can influence surveillance data and trend monitoring, we believe that this change only explains part of the increase in STEC case notifications in Switzerland.

From our analyses, indications for a real increase in STEC incidence independent of the diagnostic test method are threefold: (i) Our logistic regressions and predicted probabilities for a positive STEC test outcome showed an increasing trend between 2007 and 2016 even after adjusting for the diagnostic method, (ii) the predicted probabilities for a positive STEC test show an increasing trend for all methods (multiplex PCR, single PCR and antigen test) and (iii) an increase in positivity was also seen in two laboratories introducing multiplex PCR panels late, i.e. in the second half of 2016, or not at all. Based on these three findings, we argue that the increase in notified STEC cases is a combination of changing test practices and a real increase in incidence of STEC infections among the Swiss population.

### Rising incidence of STEC infections

Age and sex distributions of STEC patients in Switzerland remained unchanged since the observation period 2007 to 2016. We conclude that the observed incidence increase is independent of potential changes in STEC risk groups.

If our findings suggest a true increase in STEC, the epidemiology of HUS also needs to be considered. In Switzerland, the number of HUS cases remained relatively constant from 1999 to 2015 in terms of absolute numbers; hence, there was a relative decrease of HUS among notified STEC cases [12]. Thus, the increase in STEC notifications observed is likely to represent mainly mild cases and/or asymptomatic co-infections that might have been present but undetected in the past.

We propose that a changing distribution of STEC serogroups among cases could be an explanation for the change in disease severity. In other studies, O157 STEC cases were found to mostly be associated with the development of severe disease, i.e. HUS, although the importance of non-O157 infections as a cause for HUS is being increasingly recognised [32-34].

STEC culture and subsequent analysis of isolates are not routinely performed in Switzerland; the proportion of culture-based tests in our raw dataset of routinely conducted tests in 11 laboratories was only 0.1% (78/89,081, raw dataset). The scarce information on serotype distribution primarily comes from studies published by the Swiss National Reference Centre for Enteropathogenic Bacteria and Listeria (NENT) [35,36]. Analysing 2017 data, Nüesch-Inderbinen et al. [36] indicated that an isolate for further characterisation could be successfully obtained from less than 30% of multiplex PCR positive samples, suggesting limited information on serotypes in Switzerland compared with other countries. Still, using these studies and the results from research in similar contexts abroad, we can discuss the epidemiology of rising STEC incidence within Switzerland.

The two studies out of NENT reported a decrease in the proportion of STEC *stx2* carrying and *eae* carrying variants, which are both associated with severe disease in Switzerland [35,36]. Over the course of several years, the proportion of non-O157 STEC associated with human disease increased in Switzerland, other European countries and the US [35,37,38]. On the other hand, a 2013 study found that healthy people can shed *stx*-carrying bacteriophages that might lead to *stx*-positive multiplex PCR test results [39].

No EU/EEA country reported an increase in STEC notification numbers to the extent observed in Switzerland (eightfold increase, 2012–2016), except Romania, where 1 case was reported in 2012 while 29 were found in 2016 following an intensified testing after a HUS outbreak [38]. In Finland, the increase in reported cases between 2012 and 2016 was fourfold, with multiplex PCR screening introduced in 2013 [38,40]. In Norway, the notification rate increased from 0.6 to 7.6 per 100,000 population between 2007 and 2017, noting that this increase occurred mostly after 2014 and coinciding with the introduction of multiplex PCR diagnostics [41].

STEC patients associated with a recent outbreak in Finland were classified as rather mild cases [42]. The increasing STEC notifications in Norway were associated with an increasing proportion of cases classified as low-virulent while case numbers of HUS were generally constant [41]. The US also reported an increased incidence of STEC cases in 2017 compared with 2014 to 2016, although not to the extent observed in Switzerland [37]. Further, the incidence of HUS in children in the US remained similar in 2016 compared with 2013–2015, while non-O157 infections increased, resulting in a relative decrease of O157 cases. This again supports the hypothesis of an association between disease severity and serogroup, with a trend of culture-independent diagnostic tests increasing detection of less virulent strains.

Information on co-infections is neither available from the notification system nor from the data collected by the laboratories. However, up to 10% of the STEC strains obtained from clinical samples of ill individuals and identified by Nüesch-Inderbinen et al. were the same as strains isolated from the faecal samples of healthy individuals suggesting that not the identified STEC, but another pathogen was causing the symptoms [36]. This is in line with earlier reports that 3.5% of meat factory workers were asymptomatic STEC carriers [31]. In Norway, co-infections were observed in 15% of notified STEC cases detected using multiplex PCR [41]. Hence, it is likely that a minor but relevant proportion of the newly identified infections by multiplex PCR are asymptomatic co-infections.

### Implications of changing disease patterns on STEC surveillance in Switzerland

Current disease surveillance for STEC in Switzerland neither is designed to account for changes in diagnostics nor systematically distinguish between strains (particularly O157 and non-O157) that could reflect differences in virulence.

From a health systems perspective, monitoring the usage of diagnostic methods and testing algorithms applied for each notifiable pathogen among authorised and accredited diagnostic laboratories could complement surveillance data.

Since the implementation of a revised Epidemics Act in Switzerland in 2016, diagnostic laboratories are required to report the number of tests conducted for certain notifiable diseases (but excluding STEC) to the FOPH once a year. This annual reporting of summary statistics was established in the hope of improving interpretation of routine surveillance data through the incorporation of denominator data similar to that here in our study; without the need to mandate resource-intensive research for each pathogen. However, analyses of these summary statistics indicate that data quality is rather poor and that too many factors play a role to conclude on reasons for changes in test and case numbers based on summary statistics [7].

The increase of STEC cases, which are mostly mild, and the shift in serotype distribution as shown by others, changes the interpretation of STEC notifications as clinical and public health relevance needs to be considered. We believe it is critical that all cases of STEC infections, regardless of clinical relevance, are reported in order to identify clusters and sources and thus support outbreak control. However, the current effectiveness of the Swiss surveillance system for STEC could be improved incorporating strain typing information that would guide intervention and control measures, yet this also depends on achieving higher success rates of STEC isolation after PCR-positive results. The federal public health authorities recognise the need to modernise the current notification system toward electronic reporting which addresses the current issues of information availability, including more information on the diagnostic test methods used, and data inconsistency, ensuring more harmonisation between laboratory-based notifications of test results with clinical information obtained from physicians’ mandatory notifications (personal communication, Daniel Koch (FOPH), August 2019).

### Limitations

First, we selected our sample of 11 laboratories based on their contribution to the latest NNSID notifications. This choice favoured laboratories that had switched to multiplex PCR and may therefore not be representative of all laboratories in Switzerland. However, we adjusted for test method in our main trend analysis, thereby accounting for bias towards an over-representation of multiplex PCR. Second, our study only uses the actual information available to the laboratories; clinical information could not be obtained. Third, as partly evident from the data, culture-based tests and typing of STEC was very rarely performed by the participating laboratories; hence, microbiological data were not available for analysis. However, analysis of pre-existing (routine) data from laboratories can support the evaluation of surveillance data in a time- and resource-efficient manner, which could potentially be harnessed for other pathogens. Fourth, we noted that in recent years, NNSID case numbers differed from the number of positive test results recorded in the laboratories’ individual datasets. This means that positive cases were either under-reported to the NNSID, or the NNSID excluded certain reports from their official statistics or the number of positive test results in our sample was overestimated because of, for example, an insufficient exclusion of repeated tests. Finally, the correlation of laboratory, greater region and test method hampered the evaluation of spatial trends. Differences in testing and positivity rates between greater regions in Switzerland largely depend on the laboratories chosen. The differences can either relate to true differences in tests ordered by physicians between regions or they could be because the laboratories selected for our sample under-, over- or misrepresent the laboratories within their region.

## Conclusion

Since 2015, the notifications for STEC markedly increased in Switzerland. Meaningful interpretation of such surveillance data requires that every aspect of the disease trajectory, from changes in awareness (among physicians and patients) and testing behaviour to the choice of diagnostic method, are taken into consideration.

STEC surveillance has been heavily impacted by recent changes in diagnostic methods given the lack of culture-based confirmative testing and previously infrequent, but targeted testing for STEC. The switch from targeted STEC testing to co-testing of virtually all stool samples submitted for basic stool bacteriology using multiplex PCR panels has notably increased the test volume for STEC in Switzerland. However, we have found a rise in STEC cases that is disproportionally high compared to the increase in test volume, suggesting that there has been a real increase in STEC infection incidence in Switzerland.

The recently observed changes in the frequency of different serogroups and the stability of HUS cases suggests that the trend observed for STEC is mostly attributable to rather mild cases. Surveillance systems should be adapted to include information on diagnostic methods used considering the rapid development of new laboratory techniques. Modernising the notification system should also allow for a better triangulation of notified information on clinical presentation, diagnostic approaches and serotypes, provided the success rate of isolating multiplex PCR-positive samples increases.

## *Erratum

A mistake in the pathogen description was corrected on 24 August 2020.

## Supplementary Data

291000 Supplementary Material
