# Erratum for Euro Surveill. 2020;25(33)

**DOI:** 10.2807/1560-7917.ES.2020.25.34.2008271

**Published:** 2020-08-27

**Authors:** Eurosurveillance editorial team

**Affiliations:** 1 European Centre for Disease Prevention and Control (ECDC), Stockholm, Sweden

In the article ‘Do changes in STEC diagnostics mislead interpretation of disease surveillance data in Switzerland? Time trends in positivity, 2007 to 2016’ by Fischer et al. published on 20 August 2020, there was a mistake in the pathogen description at one point in the text. This was corrected on 24 August 2020.

