# Supplementary material for: Do changes in STEC diagnostics mislead interpretation of disease surveillance data in Switzerland? Time trends in positivity, 2007 to 2016
Source: Euro Surveill. 2020 Aug 20;25(33):1900584. doi: 10.2807/1560-7917.ES.2020.25.33.1900584 (PMC7441602; doi:10.2807/1560-7917.ES.2020.25.33.1900584)
Supplement: Supplementary Material [file 19-00584_MAUSEZAHL_Supplementary_Material.pdf]

SUPPLEMENTARY MATERIAL

This supplementary material is hosted by *Eurosurveillance* as supporting information alongside the article "Do changes in STEC diagnostics mislead interpretation of disease surveillance data in Switzerland? Time trends in positivity from 2007 to 2016." on behalf of the authors who remain responsible for the accuracy and appropriateness of the content. The same standards for ethics, copyright, attributions and permissions as for the article apply. Supplements are not edited by *Eurosurveillance* and the journal is not responsible for the maintenance of any links or email addresses provided therein.

Supplement S1. Seasonality of STEC testing and as a determinant for a positive test outcome

The positivity of STEC testing shows a strong seasonality. The seasonality of the total number of tests and the number of positives was calculated as the average number of tests (positives) of all test years (2007-2016) per calendar month. The number of total tests performed increased by 68% from February with 553 tests until September with 928 tests. The number of positively tested cases follows a similar seasonal pattern with 6 cases detected in February and 16 in August. Positivity peaked in July with 1.9%.

The seasonality has been incorporated into the mixed effect logistic regression using a sine and cosine functions, in the form of $\sin(d * 2 * \pi/T)$ and $\cos(d * 2 * \pi/T)$, whereas d is the time period (e.g. January, February) and T is one year, as described by Stolwijk, A. M., et al. [1]. The predicted probabilities for a positive test outcome of the univariable logistic regression are shown in Figure 1.

Supplementary Figure S1. Predicted probabilities with 95% confidence intervals per calendar month for a positive test outcome of an STEC infection for the univariable model using sine and cosine functions, 2007-2016, Switzerland

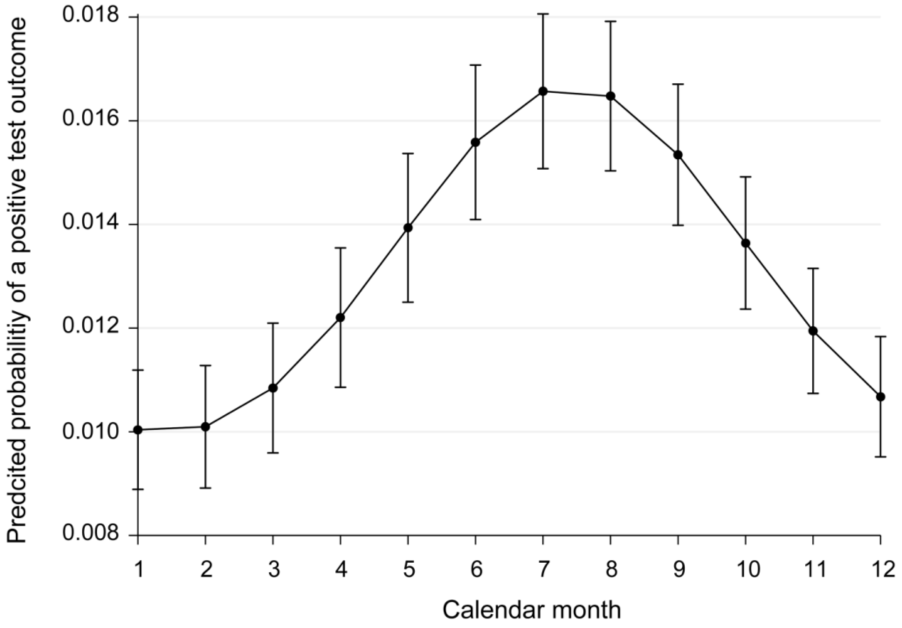

Reference

1. Stolwijk AM, Straatman H, Zielhuis GA. Studying seasonality by using sine and cosine functions in regression analysis. J Epidemiol Community Health. 1999;53(4):235-8.

Supplementary Table S1. Age and sex of the population tested for STEC and of cases with a positive result over the entire study period, 2007-2016, Switzerland

	**2007-2016**	**2007**	**2008**	**2009**	**2010**	**2011**	**2012**	**2013**	**2014**	**2015**	**2016**	**P-value test for trend**
Tested												
**Median age [years; (n)]**	40 (86'043)	30 (3'711)	29 (3'978)	31 (3'421)	29 (2'536)	31 (3'393)	37 (4'483)	42 (6'152)	43 (10'246)	44 (21'484)	43 (26'639)	<0.01
Male	38 (38'209)	28 (1'705)	29 (1'872)	29 (1'618)	26 (1'177)	28 (1'598)	33 (2'027)	39 (2'668)	41 (4'489)	42 (9'373)	41 (11'682)	<0.01
Female	42 (47'834)	31 (2'006)	30 (2'106)	32 (1'803)	31 (1'359)	33 (1'795)	40 (2'456)	45 (3'484)	45 (5'757)	46 (12'111)	46 (14'957)	<0.01
**Proportion of females [%]**	55.6	54.1	52.9	52.7	53.6	52.9	54.8	56.6	56.2	56.4	56.2	<0.01
STEC-positive												
**Median age [years; (n)]**	36 (1'149)	23 (33)	33 (31)	25 (31)	23 (15)	16.5 (38)	37 (47)	30 (84)	40.5 (126)	38 (304)	41 (440)	<0.01
Male	35 (518)	2 (11)	41 (13)	25 (17)	12.5 (6)	4 (19)	34.5 (18)	27 (46)	40.5 (56)	38 (134)	37 (198)	<0.01
Female	38 (631)	25 (22)	27.5 (18)	25.5 (14)	27 (9)	34 (19)	37 (29)	30 (38)	41.5 (70)	38 (170)	43 (242)	<0.01
**Proportion of females [%]**	54.9	66.7	58.1	45.2	60.0	50.0	61.7	45.2	55.6	55.9	55.0	0.75

Supplementary Figure S2. Row percentages of the number of STEC tests performed by test method and laboratory, 2007-2016, Switzerland.[1]

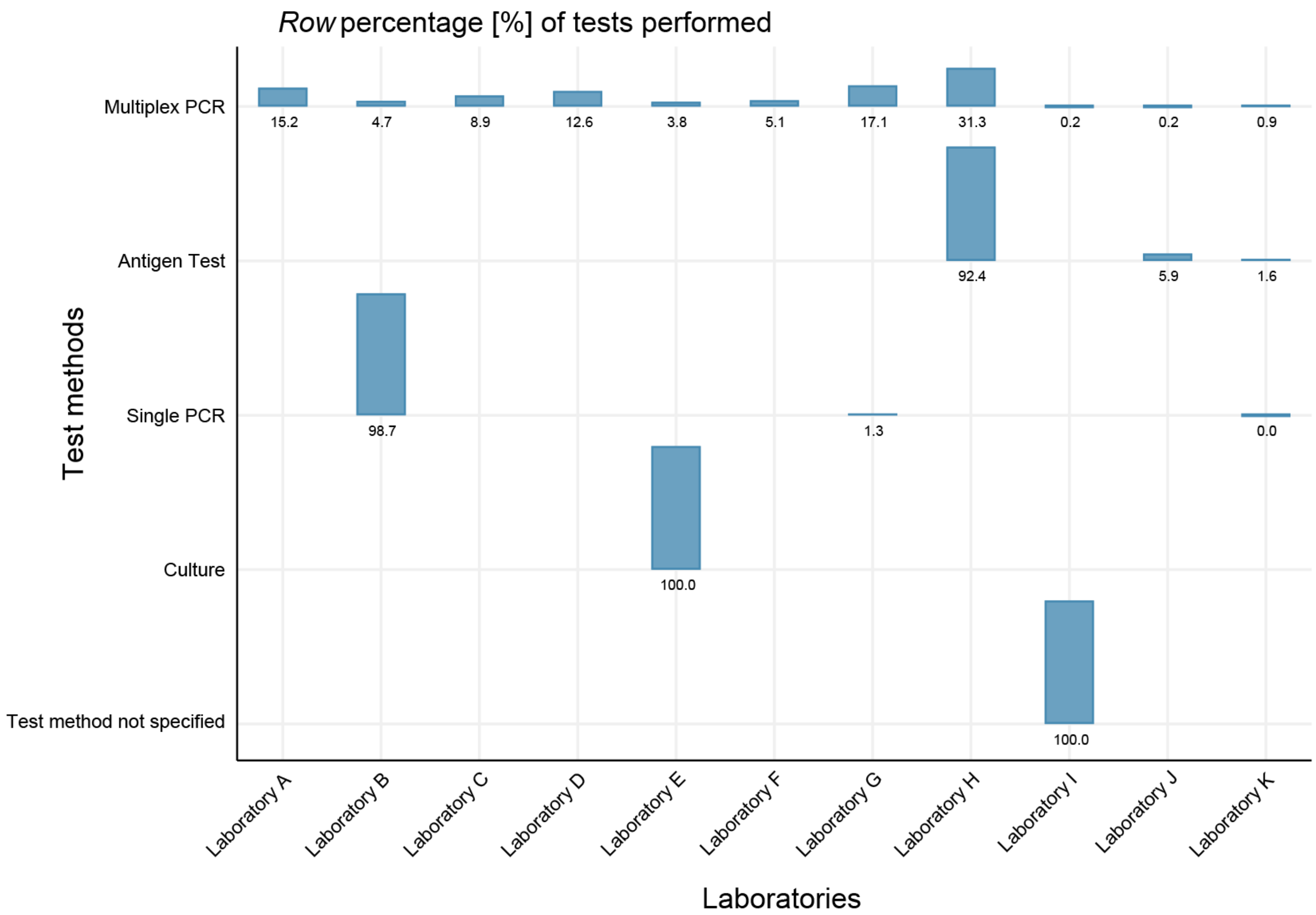

[1] The information on the correlation of greater region and laboratory is purposively omitted to ensure the anonymity of the selected laboratories.

Supplementary Table S2. Overview of diagnostic methods performed for STEC as provided by participating laboratories, 2007-2016, Switzerland. PCR panels targeting STEC/pathogenic E. coli only are referred to as 'single PCR' in contrast to 'multiplex PCR'.

**Method**	**[%]**	**Details**	**[N]**	**[%]**
**Multiplex PCR**	66.45	BD MAX™ (Extended) Enteric Bacterial Panel	29'514	34.30
		BioFire FilmArray™ *Gastrointestinal Panel*	3'368	3.91
		Luminex xTAG® Gastrointestinal Pathogen Panel	20'610	23.95
		Seegene (not specified whether Allplex™ Gastrointestinal Panel or Seeplex®Diarrhea ACE Detection) and in house method	2'629	3.06
		Multiplex PCR, not further specified	1'047	1.22
**Antigen Test**	26.26	Premier® STEC	1'341	1.56
		NOVITEC® Verotoxin ELISA	20'882	24.27
		Antigen test not specified	365	0.42
**Single PCR**	7.26	PCR after MacConkey culture	6'165	7.17
		PCR performed by external laboratory	2	<0.01
		PCR, not further specified	80	0.09
**Culture**			24	0.03
**Samples sent to NENT**			16	0.02
				100.00

Legend: NENT, Nationales Zentrum für enteropathogene Bakterien und Listerien (National Reference Centre for Enteropathogenic Bacteria and Listeria)
